# Bone Health in Idiopathic Inflammatory Myopathies: Diagnosis and Management

**DOI:** 10.1007/s11926-021-01016-8

**Published:** 2021-07-01

**Authors:** Anett Vincze, János Gaál, Zoltán Griger

**Affiliations:** 1 Division of Clinical Immunology, Faculty of Medicine, University of Debrecen, Móricz Zsigmond út 22, Debrecen, H-4032 Hungary; 2 Gyula Petrányi Doctoral School of Clinical Immunology and Allergology, University of Debrecen, Debrecen, Hungary; 3 Department of Medicine, Kenézy Gyula University Hospital, University of Debrecen, Debrecen, Hungary

**Keywords:** Osteoporosis, bone, fracture risk, Vertebral fractures, Myositis, Glucocorticoid

## Abstract

**Purpose of Review:**

This article provides an update on the most recent advances in epidemiology, pathogenesis, diagnostic procedures, and therapeutic approaches for myositis-associated bone diseases, such as osteoporosis and bone fractures.

**Recent Findings:**

In the recent years, several studies showed that osteoporosis and consequent fractures are a common and frequently underestimated complication in patients with idiopathic inflammatory myopathies (IIM). In younger patients, asymptomatic fractures might present in the early phase of the disease which could increase the risk of development of further fractures. High-risk patients could be selected with early application of combined diagnostic procedures, such as fracture risk scores with steroid dose adjustments and imaging.

**Summary:**

Recent advances might help clinicians from different fields of medicine in the early recognition and management of myositis-associated osteoporosis, which will potentially improve the quality of life of patients with IIM.

## Introduction

Idiopathic inflammatory myopathies (IIMs) are heterogeneous systemic disorders, affecting muscles, skin, or lungs, which include the subtypes of polymyositis (PM), immune-mediated necrotizing myopathy (IMNM), dermatomyositis (DM), amyopathic dermatomyositis (ADM), juvenile dermatomyositis (JDM), and inclusion body myositis (IBM) [1]. The immediate priority for clinicians treating patients with IIM is managing the disease, particularly the constant muscle weakness, interstitial lung disease, inflammatory arthritis, and skin manifestations, which might be related to significant morbidity and mortality. However, IIMs are frequently associated with systemic skeletal complications, such as osteoporosis (OP) and consequential bone fractures. The causes of fractures of patients with IIM are multifactorial: (1) the use of high doses of glucocorticoid (GC) for prolonged periods contributes to glucocorticoid-induced osteoporosis (GIOP), (2) the increased osteoclast activity caused by inflammatory mediators and immobility predispose to accelerated bone loss, and (3) the impaired mobility due to muscle weakness leads to increased risk of spontaneous falls and consequently increases bone fragility. The present review aims at summarizing the work recently published on the bone health of patients with IIM and discuss the relevant diagnostic and therapeutic interventions.

### The Pathogenesis of Osteoporosis in Idiopathic Inflammatory Myositis

**Fig. 1:**
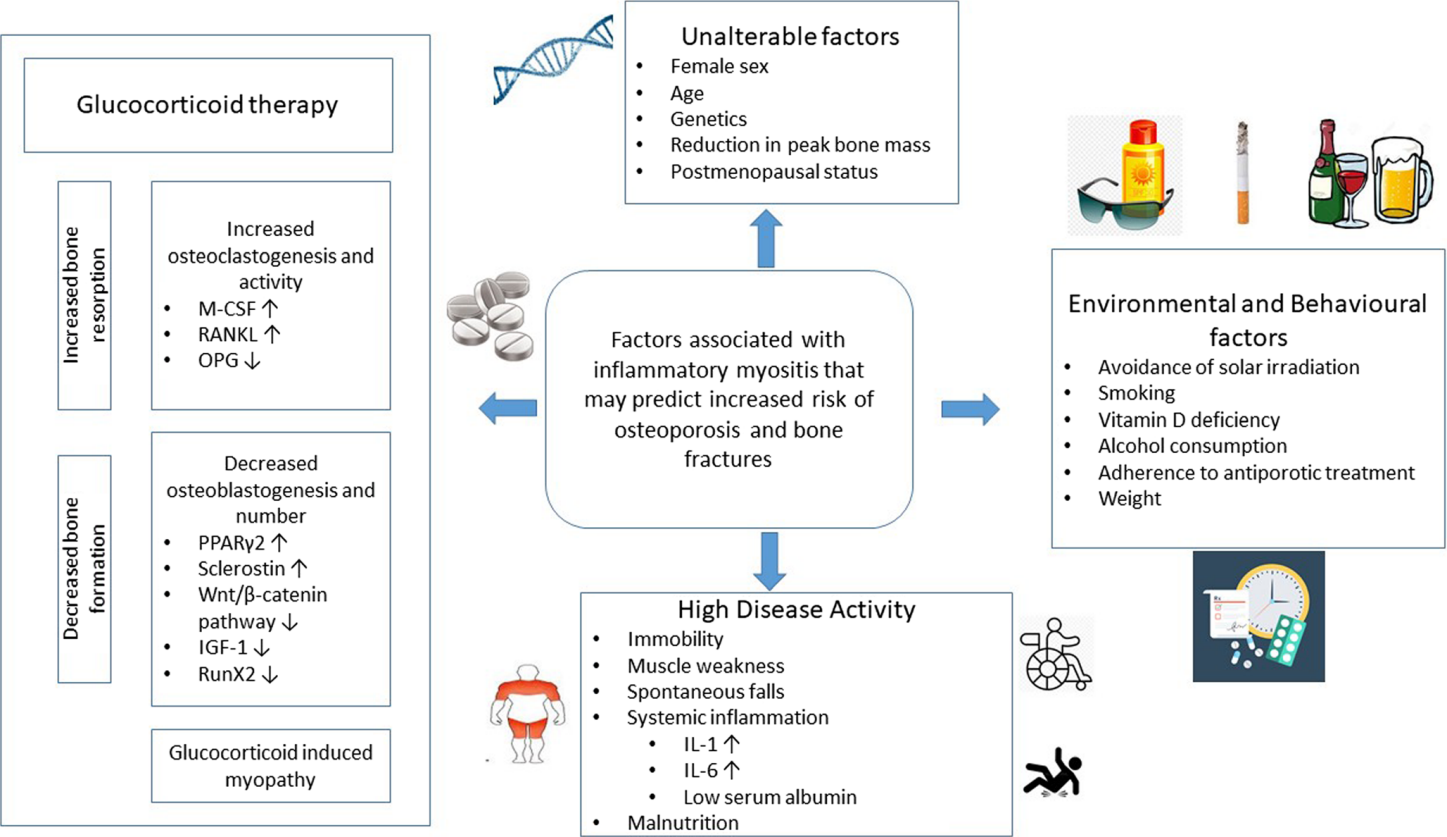
Pathogenesis of osteoporosis and fractures in IIM. M-CSF, macrophage colony-stimulating factor; RANKL, receptor activator of nuclear factor-kappa-Β ligand; OPG, osteoprotegerin; PPARγ2, peroxisome proliferator-activated receptor-gamma receptor 2; IGF, insulin-like growth factor-1; RunX2, runt-related transcription factor 2; IL-1, interleukin-1; IL-6, interleukin-6

Osteoporosis and osteopenia (the precursor of osteoporosis) are systemic diseases characterized by low bone mineral density (BMD) and reduced bone quality, resulting in an increased risk of fractures. This compromised bone strength is relevant comorbidity of IIM and is caused by the concurrence of several traditional and disease-related factors (Fig. 1). The cutaneous manifestations of DM are extremely photosensitive; thus, most of the patients are educated to avoid of UVA and UVB and to use sun protection factors, topical sunscreens, which can lead to inadequate vitamin D_3_ supply. Immobility is also an important cause of bone loss, because decreased mechanotransduction increases osteoclast function and decreases osteoblast activity, thereby shifting bone metabolism from formation to resorption. In the long term, this leads to damage to the microstructure of the bone and bone loss and ultimately to increased fragility, resulting in increased morbidity and mortality [2–4]. Inflammatory cytokines like IL-1, TNFα, and IL-6 are elevated in the sera of IIM patients [5–7], which may stimulate osteoclastogenesis while decreasing osteoblastogenesis [8].

The management of IIM has been very challenging, because it is largely based on historical clinical practice, case series, and fundamentally guided by the opinion of experts, but GCs are still the first-line drugs in the treatment of inflammatory myopathies. It is widely accepted that glucocorticoids improve disease activity in all disease subtypes except sporadic IBM, which does not respond to immunosuppressive drugs. The adverse skeletal effects of GC excess were first described over 80 years ago, and today, GC treatment is the most common secondary cause of osteoporosis [9•]. Among chronic GC users, up to 30–50% had a low energy fracture [10]. GC-induced bone loss is biphasic, beginning with an initial, rapid phase in the first 6 months, because of the high GC dose and the greater disease activity, and can reach up to 12% during the first year. This period is followed by a slower phase, where bone loss is only 2–3% per year [9•, 11, 12]. GIOP is thus fundamentally characterized by decreased bone formation, with an additional early but transient increase in bone resorption [9•]. Increased osteoclast activity mediated by macrophage colony-stimulating factor (M-CSF) and receptor activator of nuclear factor-kappa-Β ligand (RANKL) may be responsible for the increased bone resorption in the early phase together with decreasing production of osteoprotegerin (OPG) by osteoblasts and osteocytes [13, 14]. Other direct effects of GCs on bone formation are mediated through upregulation of peroxisome proliferator-activated receptor gamma receptor 2 (PPARγ2) which subsequently increases the differentiation of precursor cells to adipocytes rather than osteoblasts. Moreover, the GCs increase the expression of sclerostin and Dickkopf-related protein 1 (Dkk-1), which cause the inhibition on the Wnt/β-catenin signaling pathway, thereby additionally decreasing the number of osteoblasts. GC-evoked oxidative stress accelerates apoptosis of osteoblasts and suppresses insulin-like growth factor-1 (IGF-1), a hormone crucial for general growth and bone formation. In addition, GCs can suppress the osteoblast differentiation of mesenchymal stem cells through the induction of down-regulation of runt-related transcription factor 2 (RunX2), which also deemed to be important in the development of osteoblasts [11, 15, 16]. These processes lead to the average annual bone loss in the lumbar spine is around −2.35% and −1.95% in femoral neck [12]. Moreover, at the same bone density, patients taking glucocorticoids have a higher fracture rate than those not taking glucocorticoids. This suggests that glucocorticoids negatively affect not only bone mass but also bone quality (strength) [11]. The high risk of fracture in patients with myositis with GIOP cannot be explained by not only a decrease in bone mineral density (BMD) alone but also by a number of other factors, e.g., increased risk of falling and adverse effects on muscle mass and function, leading to GC-induced myopathy and frailty [9•, 17]. Trabecular bone in the spine is more susceptible to GC-induced loss than cortical bone [12]. Therefore, due to early accelerated trabecular bone loss, the annual incidence of vertebral fracture is around 5.1%, and non-vertebral fracture 2.5% in patients who recently started taking GC. In patients with chronic GC use, vertebral fracture is 3.2% and non-vertebral fracture is 3.0%. On long-term GC treatment, 30–50% of patients will experience an incident osteoporotic fracture [9•, 12]. Usually in patients on long-term (3 months) oral GC therapy, the threshold value considered harmful to the bone varies between 5 and 7.5 mg daily of prednisolone or equivalent [9•, 11, 12]. While all recipients of GCs are at increased risk of bone loss, older men and postmenopausal women are at the highest risk with GC doses of >20 mg daily [10]. Forty milligrams or higher prednisone equivalent a day can result in substantial BMD loss at the lumbar spine in as little as 2 months [18]. The current glucocorticoid usage has been associated with increased risk of bone loss in the hip (OR: 2.6) and spine (OR: 2.7) when followed over 2 years [15].

### The Epidemiology and Predictors of Osteoporosis and Fractures in Idiopathic Inflammatory Myositis Patients

There were only a few studies in recent years aiming at the evaluation of bone health in patients with IIMs. An overview of the most recent evidence on the presence of osteoporosis and fractures of patients with IIM is presented in Table 1*.* In the different cohort studies, the prevalence of osteoporosis was between 23.5 and 26.9%, while the prevalence of osteopenia was between 47.4 and 62.7%. The presence of both vertebral and non-vertebral fractures was found between 17.5 and 75% of the patients. In a Brazilian case-control study, it was proved that osteoporosis was more frequent in female DM/PM patients than in controls measured by DXA in both lumbar spine and the femoral neck. Moreover, a high prevalence of fractures was found in patients in comparison to healthy subjects (17.9 vs. 5.1%, *p* = 0.040; OR = 3.92; CI 95%:1.07–14.33) [23]. In a large population-based retrospective analysis from Taiwan, the authors found that patients with DM/PM were 2.99 times more likely to develop osteoporosis than those without DM/PM. After a 13-year follow-up period, the cumulative incidence for osteoporosis in the DM/PM cohort was 5.35% higher than the incidence for the comparisons. Interestingly, the osteoporosis risk was independent of corticosteroids and immunosuppressant treatment. However, some essential data was lacking, including detailed demographic information on smoking habits, alcohol consumption, body mass index, socioeconomic status, physical activity, vitamin D deficiency, calcium/vitamin D supplements, and bone-strengthening medication [22]. Data from a single-center study revealed that female gender, low serum albumin levels at onset, high Myositis Disease Activity Assessment Visual Analogue Scales (MYOACT) score, and high cumulative prednisolone dose were associated with lower BMD results [20]. Similarly, during a long-term follow-up in a single UK center, patients with long-term prednisolone doses of more than 5 mg had a significantly shorter time to develop osteoporosis/osteopenia (*p<*0.0001) than those with less than 5 mg [24]. In a more recent study by Gupta et al., in a relatively young cohort, asymptomatic vertebral fractures were present in nearly half of the patients. This was much higher than it was found from lupus patients from the same center, without ethnic and environmental differences; thus, it seems plausible that a higher fracture rate is due to disease-specific factors [21•]. Regarding the affected bones, the 11^th^ and 12^th^ thoracic vertebrae were the most commonly (30.4%) fractured. The only available longitudinal data were recently published by this group [26••]. They found in the original, but a smaller patient population that the fracture rate increased from 46 to 61.29% after 3 years. In addition, those patients who had previous vertebral fractures had a higher risk of developing a new fracture when compared with those with no vertebral fractures (76.5% vs. 14.28%, RR: 5.35). The number of fractures correlated significantly with age, T scores at the L4 level, and lower third of radius on DXA, myositis damage index (MDI), and modified MDI, where osteoporotic fracture item in MDI was removed. Neither conventional nor disease-related variables differed between progressors and non-progressors [26••]. In our Hungarian center, IIM patients with older age and longer disease duration were investigated and compared with age and gender-matched rheumatoid arthritis (RA) patients. The prevalence of osteoporosis was found to be significantly higher in the myositis group (7% vs. 13.5%, *p*: 0.045), but the fracture prevalence was similar in the two groups (75% vs. 68%) [19•]. In contrast with the data by Gupta et al., the most commonly affected vertebras were the 7^th^ and 8^th^ thoracic and the 5^th^ lumbar in this cohort (unpublished data), which might be the consequence of different age or ethnicity of the two populations. The fracture rates were independently associated with age in the myositis group, and with lower BMD results in the RA patients. Interestingly, the cumulative steroid dose was significantly higher in the myositis group but showed no correlation with the presence of vertebral fractures. The number of prevalent fractures was significantly correlated to poorer physical function detected by Health Assessment Questionnaire (HAQ) and poorer health status detected by Short Form-36 (SF36) in the myositis group [19•]. Therefore, it can be concluded that both the prevalence and the risk of osteoporosis and fractures in patients with IIM are higher than in healthy individuals and that fractures significantly affect the quality of life. The results showed a good concordance with data of groups from different regions of the world, suggesting that the high fracture prevalence is a global myositis-dependent feature. Even in younger patients, asymptomatic fractures might present in the early phase of the disease and this could increase the risk of development of further fractures.

**Table 1:** The epidemiology and predictors of osteoporosis and fractures in idiopathic inflammatory myositis patients

Reference	Country	Number of IIM patients	Mean age at examination (years)	Females (%)	Menopausal at examination (%)	BMI (kg/m^2^)	Disease duration (years)	Osteopenia (%)	Osteoporosis (%)	Factors associated with osteoporosis	Fractures (% of pts)	Factors associated with fractures
Vincze et al. (2019) [19•]	Hungary	52	57.46	82.7	76.67	26.39	13	60	13.5		75	(+) age
												(-) physical function (HAQ)
												(-) Quality of life (SF36)
So et al. (2016) [20]	China	38	52.8	84.2		23.8	4.7	47.4	23.7	(+) female sex		
										(-) serum albumin		
										(+) cumulative steroid dose		
										(+) Disease activity (MYOACT)		
Gupta et al. (2018) [21•]	India	100	35.5	82	23.5	22.2	3	62.7	26.9		46	(+) age
												(+) postmenopausal years
												(-) T and Z scores at the lower third of the radius
Wei-Sheng Lee (2016) [22]	Taiwan	1179	43.9	65.4						(+) DM/PM history	17.5	(+) age
de Andrade et al. (2012) [23]	Brazil	40	51.93	100	77.5	27.76	9.62		25	(+) age		(-) weight
										(-) weight		(+) postmenopausal status
										(+) postmenopausal status		
Ng KP et al. (2009) [24]	UK	55	41	66			9	32.7%		(+) steroid dose		
Ponyi et al. (2005) [25]	Hungary	105	50.6	76			8.9		25			

*BMI*, body mass index; *HAQ*, Health Assessment Questionnaire; *SF-36*, Short Form-36; *MDAAT*, Myositis Disease Activity Assessment Visual Analogue Scale

### Diagnostic Tools in the Clinical Practice Evaluating Bone Health in Patient with Idiopathic Inflammatory Myopathies

The most common way to determine the amount of bone is to measure *BMD*, which is the amount of bone mass per unit volume (volumetric density), or per unit area (areal density), and can be measured in vivo by quantitative CT (volumetric BMD) or densitometry (areal BMD). The most widely used techniques are based on X-ray absorptiometry in the bone, particularly dual-energy X-ray absorptiometry (DXA) [27]. The World Health Organization and the International Osteoporosis Foundation recommend that the reference technology for the diagnosis of osteoporosis is DXA applied to the femoral neck [28]. DXA of the distal radius is the most common method for measuring BMD in peripheral bones; this mode of examination provides the most information about the cortical bone rather than trabecular bone [29]. To define osteoporosis, the WHO proposed to use the T-score, which is the difference between the measured BMD and the mean value of young adults, expressed in standard deviations (SD) for a normal population of the same gender and ethnicity, and the Z-scores, which are calculated similarly, except that the patient’s BMD is compared with an age- race- and gender-matched mean value [30]. For diagnostic purposes in postmenopausal women and men over the age of 50, the T-score is basically used, while in childhood and premenopause, the use of the Z-score is recommended [27]. The trabecular bone score (TBS) is a recently developed analytical tool, which characterizes the proportion of trabecular bone within the whole bone and may improve the ability to predict the risk of fracture. Low TBS is consistently associated with an increase in both prevalent and incident fractures that is partly independent of both clinical risk factors and areal BMD at the lumbar spine and proximal femur [27, 31]. The description of new techniques developed in recent years, such as VFA (vertebral fracture assessment) or cortical thickness map, is beyond the scope of this paper. The DXA itself has a number of technical limitations: hip DXA is hampered by obesity and is not reliable enough for hyperparathyroidism, osteomalacia, thyroid disease, and renal osteodystrophy, and spinal DXA is hampered by degenerative lesions [27]. According to the guidelines, it is recommended to perform BMD testing 1 to 2 years after initiating medical therapy for osteoporosis and every 2 years thereafter. More frequent BMD testing may be warranted in certain clinical situations. The interval between repeated BMD screenings may be longer for patients without major risk factors and who have an initial T-score in the normal or upper low bone mass range [27, 31].

#### Risk Scores

Although the risk of fracture increases progressively with decreasing bone mineral density—approximately 2-fold for each standard deviation (SD) decrease in BMD [28]—a significant proportion of fractures occur in patients without osteoporosis. Therefore, the use of risk estimation methods that take into account risk factors other than BMD has become necessary for the selection of high-risk patients with a need of therapy. Accordingly, since 2008, fracture risk assessment tool models have been made available for 64 countries in 34 languages, covering 80% of the world population. The website (http://www.shef.ac.uk/FRAX) FRAX® is a computer-based algorithm that calculates the 10-year probability of a major fracture (hip, clinical spine, humerus, or wrist fracture) and hip fracture [32]. Fracture risk is calculated from gender, body mass index, and dichotomized risk factors comprising prior fragility fracture, parental history of hip fracture, current tobacco smoking, ever use of long-term oral glucocorticoids, rheumatoid arthritis, other causes of secondary osteoporosis, and alcohol consumption. Femoral neck BMD can be optionally input to enhance fracture risk prediction. Fracture probability is computed taking both the risk of fracture and the risk of death into account. However, it has some limitations: e.g., it takes no account of dose-responses for several risk factors, history of falls, etc. and is not incorporated. Relatively simple arithmetic adjustments have been proposed, high, moderate, and low exposure to glucocorticoids, concurrent data on lumbar spine BMD, trabecular bone score, hip-axis length, falls history, immigration status, and type 2 diabetes [27].

In regard myositis, the calculation of FRAX score is recommended with the use of BMD results and adjustment to the dose of glucocorticoids according to Kanis et al. [33]. Our working group highlighted that the FRAX results of the same patient population might alter significantly using different scoring algorithms (i.e., with or without BMD results and steroid dose adjustment) and the fracture risk could be underestimated [19•]. It is important to mention that FRAX score takes into account the presence of RA as a risk factor for higher fractures, but not myositis. However, the high prevalence of osteoporosis/osteopenia and fractures detected in different myositis cohorts [19•, 21•, 26••], especially in younger patients, argue for that the FRAX score might underestimate the fracture risk. Therefore, it seems logical to consider incorporating a “myositis dependent” factor that modifies the FRAX tool and allows for a more reliable risk calculation in patients with myositis. Of course, this requires prospective studies with a larger patient population and with bone fracture endpoints.

*QFracture* (www.qfracture.org) is another algorithm to calculate fracture risk. It is based on a UK prospective open cohort study of routinely collected data from general practices that takes into account numerous risk factors and estimates the 1–10-year cumulative incidence of hip or major osteoporotic fracture [34]. Like the FRAX tool, it takes into account history of smoking, alcohol, corticosteroid use, parental history (of hip fracture or osteoporosis), and several secondary causes of osteoporosis. Unlike FRAX, it also includes a history of falls (yes/no only over an unspecified time frame) and utilizes a large number of clinical risk factors but no provision is made for BMD and the tool is not calibrated to the epidemiology of other countries [35]. The National Institute for Health and Care Excellence (NICE) has recommended the use of fracture risk assessment tools (FRAX or QFracture) in the assessment of patients, including the proposal that their use should be considered in all women age 65 years or older and men age 75 years or older. In the Scottish Intercollegiate Guidelines Network guideline (SIGN 142), QFracture is preferred and is used to provide a threshold for BMD assessment [28].

The third assessment tool is *Garvan* (www.garvan.org.au), which is based on the Australian Dubbo Osteoporosis Epidemiology Study (DOES) [36]. The output of the tool reports the risk of a larger number of fracture sites. It differs from FRAX by including a history of falls and the number of previous fragility fractures but does not include other FRAX variables such as parental history of hip fracture, secondary osteoporosis, rheumatoid arthritis, glucocorticoid use, smoking, and intake of alcohol. Reasons for the differences include the derivation of fracture probability (FRAX) rather than incidence (Garvan, QFracture), poor calibration (Garvan), and inappropriate source information (QFracture) [35].

#### Traditional Radiology

Semi-quantitative or quantitative vertebral morphometry allows the identification and correct classification of vertebral deformities. X-ray studies, depending on the type and severity of vertebral body height reduction, make it possible to identify three types of vertebral fractures: wedge-shaped (anterior), biconcave (middle), and total vertebral collapse. The more accurate identification of images of the spine for a differential diagnosis of vertebral deformities providing; therefore, a visual grading of osteoporotic vertebral fractures considered mild, moderate, or severe (the Genant criteria) [37, 38]. Vertebral morphometry is carried out on the images of lateral projections of the thoraco-lumbar spine [39•].

*Bone ultrasound* reflects an independent predictor of fracture risk and measures mainly at two sites, the phalanges of the hand and the heel. It cannot be used for the diagnosis of osteoporosis, but may be recommended for epidemiological investigations and first-level screening [39•].

Using *vertebral CT*, it is possible to measure the bone component of the fractured vertebra in detail. Quantitative computed tomography (QCT) measures volumetric integral, trabecular, and cortical bone density at the spine and hip and can be used to determine bone strength, whereas pQCT measures the same at the forearm or tibia. In postmenopausal women, QCT measurement of spine trabecular BMD can predict vertebral fractures, whereas pQCT of the forearm at the ultradistal radius predicts hip but not vertebral fractures. If several vertebrae are involved, spinal MR can be used to determine bone edema, to distinguish recent fractures from older, to identify vertebrae presenting signs of impending structural failure, and to guide the vertebral augmentation interventions [31, 39•].

#### Laboratory

Biochemical markers reveal us to permit differential diagnoses, making it possible to diagnose forms of secondary osteoporosis and with repeated measurements to determine if treatment is producing expected effect, also to evaluate patient adherence to drug treatment. In addition to basic studies, the study of biochemical markers of bone metabolism is important. Laboratory also used to genetical evaluation: polymorphism of genes encoding collagen type 1, estrogen, and vitamin D receptors has been proposed as possible genetic determinants of the risk of osteoporosis [31, 39•, 40].

### Management

#### General and Special Considerations

Preventing and properly treating osteoporosis associated with IIM is a significant challenge for the clinician. Although the principles of treatment of osteoporosis in patients with IIM and the available drugs are not fundamentally different from the treatment of postmenopausal osteoporosis, there are some aspects that require special consideration. These include esophageal and gastric involvement, which makes it difficult to use certain medications; muscle weakness, which makes it difficult to use physiotherapy; and frequent steroid use, which requires a careful choice between antipyrotic agents. In addition, the majority of IIM patients with osteopenia might have a high risk of developing fractures; therefore, accurate risk stratification should be performed with the use of risk calculation tools to determine adequate pharmacological interventions. Furthermore, the disease itself can lead to bone loss, but medications used to treat the disease (primarily glucocorticoids) can also reduce the amount and decrease the quality of bone. In this regard, we should strive to achieve the highest peak bone mass at the age of twenty to thirty, as well as to prevent bone loss and non-traumatic fractures developing on the ground of osteoporosis. The fracture risk is basically determined by the patient’s actual bone mass; therefore, achieving the highest possible peak bone mass is essential. Although the peak bone mass is predetermined by genetic factors in 70–80% [41], environmental factors are also important and affect peak bone mass by 20–30% [42, 43]. Patients should be advised to lead a healthy lifestyle, sufficient calcium, vitamin D_3_, protein intake, regular physical activity, limiting the intake of carbonated drinks, and abstaining from alcohol and smoking. Adequate calcium and vitamin D_3_ supply is of paramount importance. This can be achieved by consuming 500–1100 mg of calcium and 400–600 IU of vitamin D_3_ daily. For patients on glucocorticoid treatment, lowering the glucocorticoid dose as much as possible and the use of alternate-day administration of the drug are essential as well [44].

#### Prevention of Bone Loss and Non-traumatic Osteoporotic Fractures

The basic goal of treating osteoporosis is to prevent “osteoporotic” fractures due to low energy exposure. In this, we need to use the most of both non-pharmacological and pharmacological treatment options. It is also known that prevalent fractures significantly increase the probability of further fractures; therefore, the prevention of the first fracture (primary prevention) is of paramount importance [45]. Therefore, relevant patient educational material and patient advisory cards should be used to increase the patients’ awareness and adherence to preventive pharmacological and non-pharmacological antipyrotic treatments. The most important non-pharmacological treatment and basic interventions are the following:

1. Diet, lifestyle modification, drugs

The avoidance the malabsorption and adequate (1–1.2 g/kg/day) protein intake is important for the maintenance of musculoskeletal function and for the prevention of falls and low-energy fractures [46]. The supplementation with vitamin K, magnesium, copper, zinc, phosphorus, iron, or essential fatty acids is unnecessary, reducing caffeine intake to 4 cups of coffee per day is also recommended. Smoking cessation and limitation of alcohol consumption to no more than two drinks per day are advisable as well [47]. It is important to control the underlying disease while minimizing glucocorticoid dose, with the use of steroid-sparing drugs such as methotrexate, mycophenolate, cyclosporine, azathioprine, or targeted treatment if necessary [9•]. The use of sedative and hypnotic drugs should be minimized to decrease the tendency of falls and the medicines with potentially negative effects on bone metabolism should also be avoided if possible [48].

2. Calcium and vitamin D_3_ supplementation

The adequate vitamin D_3_ and calcium supply is the cornerstone of effective therapy. The target blood 25OH D3 level is above 30 ng/ml (75 nmol/l), to ensure this an average intake of 800–2000 IU vitamin D_3_ is required. The recommended daily intake of calcium is 1000–1200 mg, preferably with food; if this is not possible, calcium supplementation is also required, preferably in the form of calcium citrate. It is recommended to avoid calcium intake above 1200 mg due to the increased risk of side effects [47].

3. Exercise and making the environment safe

Regular weight-bearing physical activity and exercises improve muscle strength, physical performance balance, and posture and decrease the tendency of fall and increase osteoblast activity, therefore highly recommended [49]. Eliminating slippery surfaces, non-slip carpets, designing ramps instead of stairs, fitting handrails, and remodeling bathrooms also reduce the likelihood of falls and fractures [49].

#### Pharmacological Interventions

The currently approved options for the pharmacologic prevention and/or treatment of postmenopausal, corticosteroid, and male osteoporosis include antiresorptive drugs (bisphosphonates, estrogens, selective estrogen receptor modulators, denosumab) and osteoanabolic drugs (teriparatide, abaloparatide, romosozumab). *Bisphosphonates* cause apoptosis of osteoclasts by inhibiting cellular metabolism; aminobisphosphonates primarily inhibit protein prenylation. The different bisphosphonates used in clinical practice (alendronate sodium, risedronate, ibandronate sodium, zoledronic acid) reduce the incidence of spine and hip fractures by about 25–70 % over 2–4 years in patients with osteoporosis; the magnitude of the fracture-reducing effect varied according to the degree of bone loss and the prevalent fractures [50–55]. *Estrogens* inhibit bone resorption by stimulating the apoptosis of osteoclasts and suppressing the apoptosis of osteoblasts, but reported potential side effects (increased risks of myocardial infarction, stroke, invasive breast cancer, pulmonary emboli, and deep vein thrombosis) should be noted [56]. The selective estrogen receptor modulator *raloxifene* is a synthetic estrogen receptor ligand, which induces osteoclast apoptosis. Its particular advantage is that it reduces the chances of developing breast cancer in the postmenopausal population [57]. *Denosumab* is a fully human monoclonal antibody against RANKL that reduces the incidence of vertebral fractures by about 68%, hip fractures by about 40%, and non-vertebral fractures by about 20% after 3 years of treatment [58].

*Teriparatide* is a synthetic parathormone fragment (PTH1-34), which activates the Wnt/β-catenin pathway in osteoblasts, thereby increasing osteoblast differentiation and proliferation. It is indicated for treatment of patients taking long-term glucocorticoid treatment. After a treatment period of 18 months in average, teriparatide reduces the risk of vertebral fractures by about 65%, non-vertebral fractures by about 53%, and hip fractures by about 65% in patients with osteoporosis [59, 60]. *Abaloparatide* is a synthetic analog of parathyroid hormone–related peptide (PTHrP), which increases osteoblast differentiation and proliferation like teriparatide, and reduced the incidence of new vertebral fractures by 43% and non-vertebral fractures by 86%, in patients with osteoporosis [61].

*Romosozumab* is a monoclonal antibody that blocks the effects of the protein sclerostin and works mainly by increasing new bone formation. Romosozumab reduced the incidence of new vertebral fractures by 75%; however, it did not significantly reduce the risk of non-vertebral fractures over 12 months of treatment in patients with osteoporosis [62].

#### Treatment Strategy

The underlying goal of antipyrotic therapy of patients with IIM is to prevent fractures. This is why it is important to select those patients who have the highest risk of fracture, as these patients can benefit the most from treatment. All IIM patients with decreased bone density should be provided with a daily intake of 800–2000 IU D3 and at least 1000 mg of calcium, and specific antipyrotic therapy for patients at high risk of fracture. In principle, patients who have already undergone a fracture and/or whose bone density reaches the level of osteoporosis (T-score≤-2,5) are considered to be at high risk. Among patients with osteopenia, the FRAX risk calculation tool can be used to find the high-risk patients (10-year probability of a hip fracture ≥3 % or a 10-year probability of a major osteoporosis-related fracture ≥20 %), those who are more prone to fractures, and also those who require treatment [31, 63].

It is practical to use oral bisphosphonates as initial treatments in the majority of cases because they are inexpensive and proven to be effective. In patients with intolerance/contraindication, intravenous bisphosphonates or denosumab serves as appropriate alternatives or raloxifene, or menopause hormone therapy in selected cases. Anabolic treatments (teriparatide, abaloparatide, romosozumab) should be considered first-line therapy in patients with prior fragility fractures and a very low bone mineral density (T-score below −3.0) especially if the fracture occurred within 2 years [64•]. Anabolic therapies can only be continued for a limited period of time (teriparatide and abaloparatide for only 2 years, romosozumab for 1 year), so after the discontinuation of anabolic therapy, bone gain can be maintained with sequential antiresorptive treatment [65].

Considering that the majority of IIM patients receive glucocorticoid therapy for longer or shorter periods of time, steroid-related osteoporosis deserves special attention. It has been shown that those taking glucocorticoids are more likely to suffer fractures at the same bone density than those not taking glucocorticoids. Therefore, long-term steroid treatment has been incorporated into the FRAX calculator as a stand-alone risk factor as a dichotomous variable (if there is current exposure to oral glucocorticoids or past exposure for ≥ 3 months at a dose of 5 mg/day or more of prednisolone or equivalent). Although the basic strategies to prevent and treat glucocorticoid-induced osteoporosis are similar to those used to manage osteoporosis due to other causes, the American College of Rheumatology recommends the administration of antipyrotic agents to adults for moderate risk as well. According to this, patients aged ≥40 years with glucocorticoid-adjusted FRAX 10-year risk for major osteoporotic fracture of 10 to 19% or risk for hip fracture of >1 to <3% and patients aged <40 years with a hip or spine bone mineral density Z-score of <−3 or rapid bone loss of ≥10% at the hip or spine over 1 year and are on glucocorticoid treatment at ≥7.5 mg/day for ≥6 months should be treated with specific antipyrotic agents [66].

## Conclusions

The heterogeneous clinical spectrum of IIMs results that the disease treatment is often guided by clinicians from different fields of medicine. Recent publications highlighted that a unique and general feature of this heterogeneous disease is the presence and significance of osteoporosis and bone fractures. Therefore, bone health and fragility should be screened with combined techniques including risk scores, imaging, and laboratory at the assessment of the diagnosis of IIM. Accurate case risk identification is important to ensure primary and/or secondary prevention of osteoporosis and fractures, which might increase the time of the patients with myositis in good quality of life.
